# Brain dynamics of (a)typical reading development—a review of longitudinal studies

**DOI:** 10.1038/s41539-020-00081-5

**Published:** 2021-02-01

**Authors:** Katarzyna Chyl, Gorka Fraga-González, Silvia Brem, Katarzyna Jednoróg

**Affiliations:** 1 Laboratory of Language Neurobiology, Nencki Institute of Experimental Biology, Polish Academy of Sciences, Warsaw, Poland; 2 Department of Child and Adolescent Psychiatry and Psychotherapy, Psychiatric Hospital, University of Zurich, Zurich, Switzerland; 3 Neuroscience Center Zurich, University of Zurich and ETH Zurich, Zurich, Switzerland; 4 MR-Center of the Department of Psychiatry, Psychotherapy and Psychosomatics and the Department of Child and Adolescent Psychiatry and Psychotherapy, Psychiatric Hospital, University of Zurich, Zurich, Switzerland

**Keywords:** Dyslexia, Reading

## Abstract

Literacy development is a process rather than a single event and thus should be studied at multiple time points. A longitudinal design employing neuroimaging methods offers the possibility to identify neural changes associated with reading development, and to reveal early markers of dyslexia. The core of this review is a summary of findings from longitudinal neuroimaging studies on typical and atypical reading development. Studies focused on the prediction of reading gains with a single neuroimaging time point complement this review. Evidence from structural studies suggests that reading development results in increased structural integrity and functional specialization of left-hemispheric language areas. Compromised integrity of some of these tracts in children at risk for dyslexia might be compensated by higher anatomical connectivity in the homologous right hemisphere tracts. Regarding function, activation in phonological and audiovisual integration areas and growing sensitivity to print in the ventral occipito-temporal cortex (vOT) seem to be relevant neurodevelopmental markers of successful reading acquisition. Atypical vOT responses at the beginning of reading training and infant auditory brain potentials have been proposed as neuroimaging predictors of dyslexia that can complement behavioral measures. Besides these insights, longitudinal neuroimaging studies on reading and dyslexia are still relatively scarce and small sample sizes raise legitimate concerns about the reliability of the results. This review discusses the challenges of these studies and provides recommendations to improve this research area. Future longitudinal research with larger sample sizes are needed to improve our knowledge of typical and atypical reading neurodevelopment.

## Introduction

Reading is one of the most important skills acquired during the first years of schooling. For the majority of children, learning how to read is fairly effortless, but around 10% show unexpected difficulties in attaining typical levels of reading skills. Developmental dyslexia is a heritable, specific learning disorder that results in persistent impairment in reading and spelling not solely accounted for by mental age, obvious sensory or neurological damage, or inadequate schooling^1^. Understanding the relationships between behavioral symptoms and neuronal changes is still a challenge for the field^2^. Dyslexia is typically not diagnosed until a child has failed to learn to read as expected, which is usually in the second grade or later. At the same time, early intervention in kindergarten or first grade for children identified as being “at-risk” has been proven to be the most effective in ameliorating later effects^3^. Therefore, a developmental framework is considered most suited to study developmental disorders. It implies that both normal and abnormal development is progressive and rather than concentrate on the study of disorders solely at their end state in school-aged children and adults, they should be examined longitudinally. Such a framework can aid in understanding how alternative developmental pathways might lead to different phenotypical outcomes.

Longitudinal studies tracking brain development with child-friendly neuroimaging techniques during the first years of reading acquisition are critical to characterize variations in the developmental trajectories of brain networks and to relate such variations to children’s reading predisposition and attained reading level. Neuroscientific studies of reading hold the promise of identifying and characterizing early risk factors that cannot be detected by cognitive assessments in pre-reading stages^4^. To advance the field of dyslexia research and clinical practice, studies recruiting pre-readers or beginning readers and tracking them until the age when dyslexia is diagnosed are especially valuable. Such longitudinal designs enable observation of neuronal alterations at or before reading onset, not affected by the limited reading experience, to reveal early markers of dyslexia. Only then is it possible to disentangle causes from consequences of dyslexia and their neurobiological basis^5^. Compared with a cross-sectional design, which currently dominates in the field, the longitudinal approach reduces the confounding effect of between-subject variability^6^, enables assessment of the predictive value of different measures, and reveals how specific neural changes are related to age and changes in reading performance^7^.

**Fig. 1:**
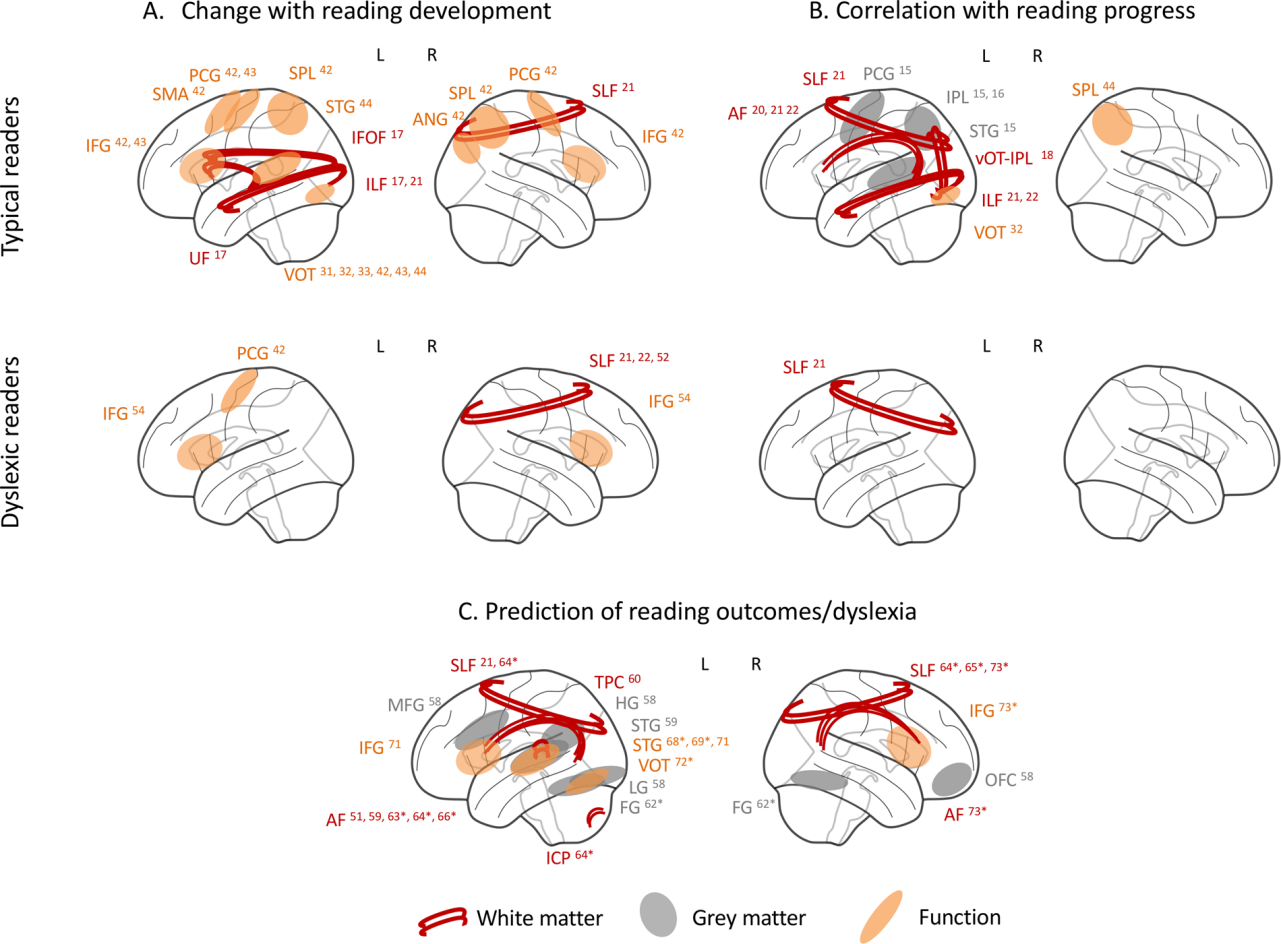
Schematic of key structures reported in longitudinal studies of reading and dyslexia. **a** Brain regions showing changes in longitudinal comparisons. **b** Longitudinal changes that were correlated with reading development. **c** Regions that predict future reading skills. Numbers indicate references summarized in Supplementary Data and asterisk indicates prediction studies with only one neuroimaging time point. NOTE: Numbers correspond to references and summaries included in Supplementary Data. Asterisks (*) mark the studies with one neuroimaging time point predicting reading development.White matter: AF Arcuate Fasciculus, IFOF Inferior Fronto-Occipital Fasciculus, ICP Inferior Cerebellar Peduncle, ILF Inferior Longitudinal Fasciculus, SLF Superior Longitudinal Fasciculus, TPC Temporo-Parietal Cortex, UF Uncinate Fasciculus. Grey matter/function: ANG Angular, FG Fusiform Gyrus, HG Heschl Gyrus, IFG Inferior Frontal Gyrus, IPL Inferior Parietal Lobule, L Lingual Gyrus, OFC Orbito-Frontal Cortex, PCG Precentral and Postcentral Gyri, STG Superior Temporal Gyrus, SPL Superior Parietal Lobule, vOT ventral Occipito-temporal Gyrus.

**Fig. 2:**
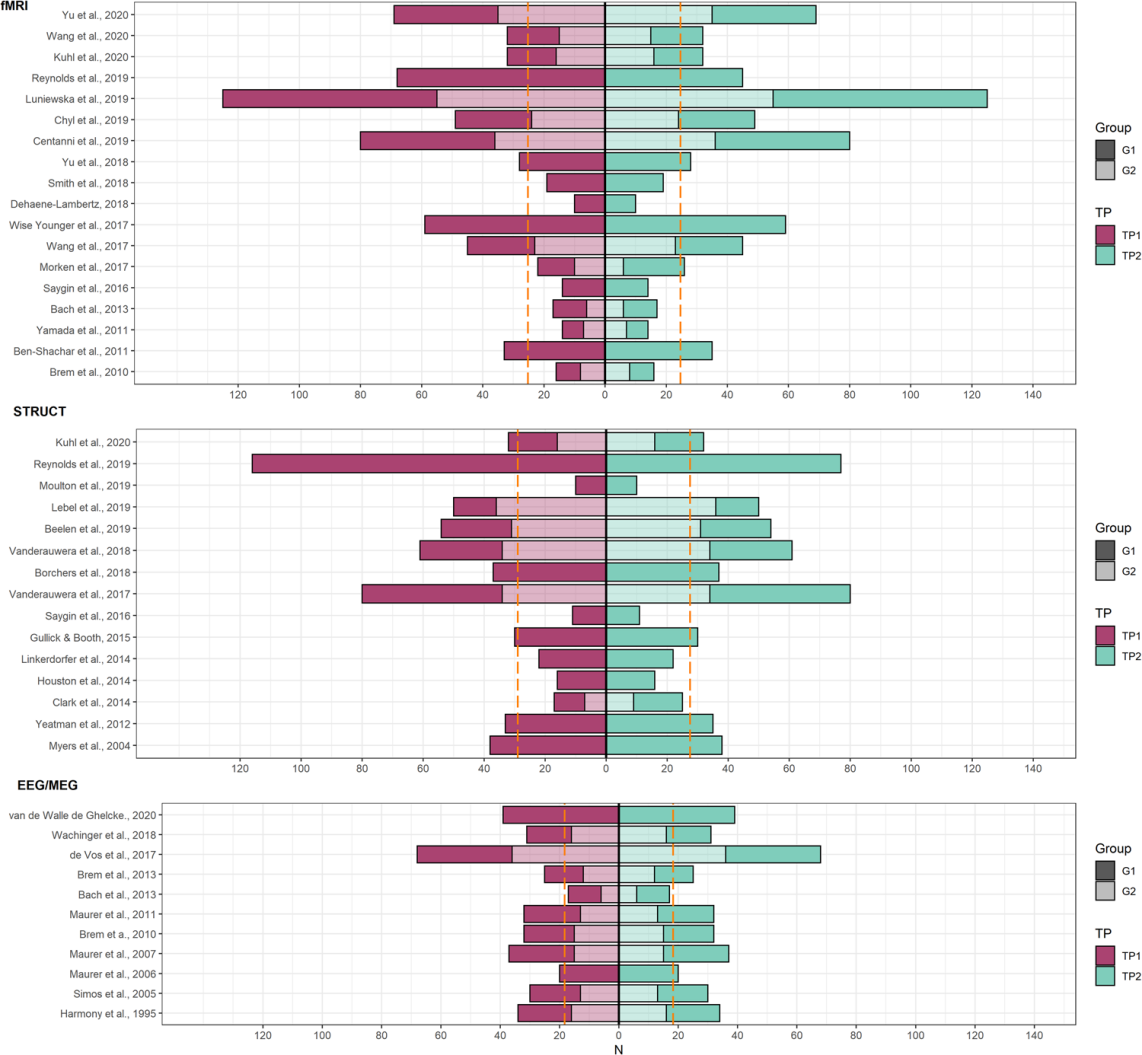
Illustration of sample sizes of studies with longitudinal EEG, fMRI and structural (WM, GMV) neuroimaging data. NOTE: Dashed orange lines indicate mean sample size across groups for a measurement time point. The darker portion of the bar indicates the control group and the lighter portion indicates the subsample of dyslexic, poor readers or at risk group depending on the study. Left-oriented bars correspond to the first time point and right-oriented bars to the second time point. For clarity purposes, only samples for two time points and sample sizes in the comparisons with the largest groups are displayed.

This review aims to discuss longitudinal studies in individuals with dyslexia and typically reading controls that employed neurophysiological measures such as structural and functional magnetic resonance imaging (MRI), and electroencephalography (EEG). It concentrates on studies presenting neuroimaging data of at least two time points. First, we will review the most relevant findings on typical reading development to establish a foundation for discussing the neural basis of dyslexia. These studies aim at differentiating brain changes associated with learning how to read from general maturational effects. This section also refers to several training studies and studies investigating groups of at-risk and/or poor readers, as they clarify the functional role of the brain networks implicated in reading. Next, the main section of this review summarizes longitudinal evidence for atypical neural development in dyslexia and discusses whether it supports a developmental delay or rather, specific alterations, as two competing hypotheses to explain the neurobiology of dyslexia. Since the scope of this review is limited, and the focus is on general reading development, studies assessing brain changes after interventions for dyslexia are not discussed. The last section focuses on early prediction and comments on the feasibility of using neuroimaging measures as markers for early diagnosis. This section is complemented by references to several studies that only used one neuroimaging data time point to predict gains in longitudinally assessed reading skills (for other comprehensive reviews that cover this literature see refs. ^8–11^). A schematic of the main brain areas reported in this review is presented in Fig. 1 and the summary of longitudinal studies of typical reading and dyslexia are presented in Supplementary Data. This table also includes a synopsis of results and the most relevant demographic information on the participants. Figure 2 provides the sample sizes of the reviewed studies, as the low number of participants is particularly problematic in longitudinal pediatric neuroimaging due to the possible dropouts that further limit the reliability of retrospective findings. Finally, we discuss the challenges involved in longitudinal designs to study dyslexia and propose recommendations to improve this research area.

## Typical reading development

### Brain structure

Recent studies with rigorous data quality control showed that brain maturation involves dynamic changes with increases in white matter volume as well as decreases in gray matter volume (GMV) over time from childhood to adolescence. Cortical thickness also declines gradually after the age of 5, and only bilateral temporo-parietal areas and the right prefrontal cortex peak at age 8 and decline from then on^12–14^.

In the context of reading development, gains in reading were positively correlated with GMV in the left superior temporal gyrus (STG) at the age of 7.5^15^, a peak in the trajectory of GMV growth in this area^12^. In the same cohort examined a year later, better reading skills correlated with stronger decreases of GMV in the left inferior parietal lobule as well as in the precentral and postcentral gyri^15^. In another study using a wider age range (initially 5–15-year olds scanned twice in the course of two years), GMV decrease in parietal cortical and inferior frontal regions was also associated with better word decoding, reading fluency, and rapid automatized naming^16^. Thus, it seems that dorsal frontoparietal circuit maturation, implicated in the integration of phonology with orthography as well as attention, is particularly important for reading development. However, the causal direction of this relationship is still unclear, since faster maturation may be a consequence rather than a cause of reading progress.

Studies examining white matter have mainly used the measure of fractional anisotropy (FA). This measure is thought to reflect properties such as fiber density and myelination, and it is proposed as an index of white matter integrity. A shift from the pre-reading (5–6 years) to the early reading stage (7–8 years) was paralleled by an increase in FA of several ventral white matter pathways (inferior fronto-occipital fasciculus, inferior longitudinal fasciculus (ILF), and uncinate fasciculus)^17^. Moreover, microstructural changes (decreased radial diffusivity related to increased myelination) in the ventral connections between the visual word form area (VWFA) and left inferior parietal cortex correlated with improvements in reading scores over the first year of reading instruction^18^. Besides these ventral tracts, an important dorsal tract, that studies have often focused on, is the arcuate fasciculus (AF), which links the lateral temporal cortex with the frontal cortex and is related to language^19^. Leftward FA asymmetry of the AF increased across a 2–7.5 year age range. Although this change was not related to pre-reading phonological skills, the left AF microstructure correlated with pre-reading skills^20^. In another study, the rates of FA development in the left AF and ILF from the pre-reading to fluent reading stage were found to be positively correlated with improved reading abilities^21^. In line with this observation, children with above-average reading skills initially presented low FA of the left AF and ILF that increased over a 3 year period^22^. Interestingly, children at risk for dyslexia who became good readers also showed a faster rate of FA development in the right superior longitudinal fasciculus (SLF, connecting the parietal, occipital, and temporal lobes with ipsilateral frontal cortices) than those who developed into poor readers^21^. Thus, the alterations in the integrity of left hemisphere tracts that contribute to typical reading development before reading onset in individuals at risk for dyslexia might be potentially compensated by high integrity of the homologous tracts of the right hemisphere.

These studies suggest that age-appropriate GMV decrease and faster white matter maturation may indicate typical reading development. These changes are thought to reflect both an increase in myelination and axonal diameter as well as synaptic pruning^23^. The maturational trajectories of gray and white matter networks can be considered in relation to cognitive development^24^ and specifically to reading acquisition. When reviewing and interpreting studies on reading and brain structure, it is important to remember that maturational trajectories of gray and white matter differ between the regions^13^.

### Brain function

The development of the function of the human brain begins prenatally with synchronized oscillatory network activity believed to be essential for the generation of neuronal cortical circuits^25^. Long before any effective language production, infants show high competence in their receptive language skills and adult-like brain responses^26^. Structural maturation, in particular myelination, probably interacts with and facilitates the experience-dependent postnatal development of spatially distributed functional networks^27^.

The acquisition of reading skills involves functional reorganization of sensory and associative brain systems that become implicated in language processing^28–30^. In particular, the specialization of ventral visual systems to print has been widely studied as a key step to successful reading fluency development. An fMRI study showed that left ventral occipito-temporal (vOT) sensitivity for printed words could emerge after just a few hours of grapheme–phoneme correspondence training in 6-year olds^31^. Growing sensitivity of this area during reading development was also demonstrated in elementary school children across four measurement times^32^. In that study, cortical sensitivity to print in the left vOT (but not in V1 or the right vOT) was correlated with age and, more importantly, with the rate of improvement in reading^32^. The process of shaping the VWFA in the left vOT was recently examined in a small group of 6-year olds scanned 6 or 7 times before and throughout the first year of school. Along with reading development, voxels previously weakly specialized for object processing became specifically tuned to written words and digits^33^ supporting neuronal recycling hypothesis^34^. Additionally, the location of the VWFA at the age of 8 could be predicted by the pre-existing structural connectivity from this region to the left-hemispheric language areas already present at the pre-reading stage^35^.

Several event-related potential (ERP) studies have focused on the electrophysiological correlate of VWFA activity, i.e., a negative potential (N1) over occipito-temporal scalp regions with latencies around 150–220 ms associated with visual expertise^36–38^. Longitudinal data suggested a non-linear trajectory for a coarse N1 sensitivity to words vs symbol or false font strings that emerged during the transition from the pre-reading to the reading stage^36,37^ (i.e. from kindergarten/preschool to second grade in children), which leveled off with more practice from second to fifth grade^38^. In a shorter time frame, a longitudinal study found emerging N1 sensitivity to print in kindergarten after a few hours of grapheme–phoneme association training over a period of around 8 weeks^31^. These findings were complemented by reports of more subtle N1 amplitude discrimination and faster processing in adult readers^39^. Specialization in the left vOT was further investigated in a recent EEG study using a fast periodic stimulation paradigm and examining 6-year-old beginning readers before and after 1 year of schooling^40^. In this study, letter strings or words were inserted periodically in sequences of pseudofonts or pseudowords, respectively. Letter string selective responses increased with time and were detected in both measurements. This suggests a very early onset of visual tuning for letter strings. However, the study found no sensitivity to words in pseudoword condition in any of the measurements, probably reflecting a lack of automatization in visual word decoding in children. Altogether, the evidence from visual electrophysiological responses suggest an inverted U-shape development of visual specialization to print with reading expertise. This observation has motivated an interactive account of the vOT function^41^ that emphasizes the dynamic contribution of top-down predictions from other language areas (involved in phonology and semantics) across reading development. According to this model vOT activation is the highest during learning, when the stimulus is recognized as potentially meaningful, but with a high prediction error. The prediction error decreases with higher expertise leading to a decline in vOT activation.

In addition to visual areas, increased activation to print in typical readers after two years of reading instruction was also reported in regions of the language network, i.e., bilateral inferior frontal gyri (IFG) and precentral gyri (PrCG), left supplementary motor area (SMA), bilateral superior parietal lobule, and right angular gyrus^42^. To assess whether or not these effects follow an inverted U-shape development similar to vOT would require further studies tracking reading development later than second/third grade. In another study, growing reading expertise was also related to decreasing functional connectivity in a reading task between the left vOT and the left IFG/PrCG from the pre-reading stage (age 6), to emergent literacy (age 8), and literacy (age 12)^43^. Downregulation of connectivity could reflect that these connections are important to establish reading skill, but that once automaticity is reached, they are no longer needed, and hence taper off. A recent study showed that differential processing of audiovisual congruent and incongruent nonword information during an implicit task emerges in the second grade^44^. The strength of the developing congruency effect in the left STG from the beginning (age 7) to the emergent reading stage (age 8.5) was associated with improvements in pseudoword decoding and with an increase of functional coupling over time between the left vOT and the right superior parietal lobe for congruent audiovisually presented nonwords. These results suggest a growing involvement of the dorsal attention network for matching audiovisual information. Recent evidence from adults suggests VWFA in left vOT is tightly coupled to both language and attention networks in the brain, with language and attentional abilities reflected in their respective connectivity patterns^45^. It is possible that the strength of these connections shows a differential development depending on the reading stage and/or task which would explain these seemingly contradictory findings.

Audiovisual integration was also examined in a longitudinal ERP study, which followed children across five time points from kindergarten to the end of the second grade^46^. The analysis focused on a late positive component (LPC) over the temporo-parietal scalp electrodes elicited by an explicit reading task, where congruent or incongruent audiovisual pairs were presented with either words or pictures. The LPC amplitudes increased from kindergarten to first grade and subsequently decreased from the second to the fifth time point. This evidence suggests that these neurophysiological responses are strengthened in the initial phases of learning and tend to decline with increasing expertise and facilitated processing. Similarly, in longitudinal fMRI studies focused on phonological awareness, brain activity decline with increasing expertise was found in agreement with the Interactive Specialization framework^47^, suggesting refinement of brain pathways involved in a given cognitive function and a more focal pattern of activation with increasing age and experience. In an auditory phonological task, a developmental decrease in activation in the left inferior parietal cortex (IPC) and bilateral precuneus in typical readers from the beginning of formal education until the emergent reading stage (5 to 7–9 years of age) was reported. Another study^48^ revealed reduced activity in the left perisylvian cortex (including IPC) for the auditory rhyming task in typical readers after the first 2 years of formal education, which was more pronounced in children without familial history of dyslexia. Functional connectivity analysis revealed that the connection strength between the left IPC, left IFG, left vOT, and right angular gyrus co‐developed with the growth of phonological skills^49^. This study also found that functional connectivity in this network was strengthened over time in those participants showing above-average phonological gains, while an opposite trajectory was found in the subgroup with lower phonological gains^49^. However, in older readers aged 8–14 and scanned 2–3 years later, higher initial connectivity between the left IPC and left vOT in the visual rhyming task was shown to decrease with developmental increases in reading ability^50^. Since both the modality of the phonological task (auditory vs. visual) as well as the developmental stage could have modulated connectivity, we cannot infer if the connectivity strength of this phonological brain network shows a U-shaped development.

To summarize, typical reading development is characterized by the structural maturation and increased structural connectivity of the left language areas. The left vOT cortex becomes specialized for processing words over other visual symbols. The developmental trajectory of the vOT could be described by an inverted U-shaped function, as EEG studies suggest, with growing specialization at the shift from the pre-reading to the reading stage which levels off with higher expertise. Similar to the visual specialization to words, the few longitudinal ERP and fMRI studies on phonological processing suggest an inverted U-shaped developmental trajectory and a refined, more focal pattern for the involved processes and brain networks. However, more longitudinal studies are necessary to clarify the associated developmental alterations in more detail.

## Development of the reading network in dyslexia

### Brain structure

A different developmental pattern of white matter structural connectivity was observed in dyslexic readers as compared to age-matched peers. Already prior to the reading onset, children with dyslexia showed lower FA of the bilateral long AF segments than typical readers, but after the reading onset differences in the left AF were canceled out^51^, demonstrating that white matter deficits are dynamic and potentially influenced by the amount of reading intervention a child received. In another study, children with dyslexia initially aged 9.5 and scanned after 1.5 years did not show age-related increases of FA in several regions of interest (among them, the right SLF) present in typical readers^52^. In line with this observation, children at risk of dyslexia who developed into poor readers have shown slower rates of FA development in the right SLF^21^. In a different study, a decline of FA in the left AF and ILF over time was observed in poorer readers^22^. Another measure used to assess white matter properties is mean diffusivity (MD), representing the magnitude of water diffusion within the brain, with lower values reflecting tissue compactness and myelination. It was shown that MD of the right corona radiata and left uncinate fasciculus have steeper decreases in the dysfluent inaccurate readers than dysfluent, but accurate readers^52^. This result shows that children with dyslexia who exhibit different component skills in reading may also have different maturation trajectories, with steeper MD decreases possibly reflecting compensatory maturation in the inaccurate group^52^. Groups, however, did not differ in the correlations of reading and diffusion parameters – in both children with dyslexia and controls, reading was positively correlated with FA in the left SLF and phonological decoding was negatively correlated with MD in the corona radiata^52^. This result suggests a delay of white matter development in dyslexic children rather than an altered developmental pathway.

### Brain function

Studies interested in literacy development for a long time suggested different engagement of the reading networks in individuals with dyslexia, with deficits present both in the dorsal temporo-parietal circuit (especially at the first stages of reading development), and ventral occipito-temporal circuit deficits arising later on^53^. However, this classical model was largely based on cross-sectional findings and studies examining adults. Nonetheless, as recent longitudinal studies showed, disrupted reading development or even dyslexia risk is in fact associated with a different developmental trajectory of functional brain reorganization. Children at risk of dyslexia aged 5–6 years hypoactivated the reading network at the early stage of reading training. Three months later, after intensive reading instruction, they showed bilateral temporo-parietal and frontal activity for words, while the control group employed left-lateralized temporo-parietal regions^54^. A magnetic source imaging study showed that 6-year olds with a high risk of dyslexia showed no leftward lateralization for reading tasks, while this pattern was clear in the control group. Lack of asymmetry reflected the increased activity of the right hemisphere, rather than left hemisphere hypoactivation, and was stable across kindergarten and first grade^55^. Similarly, an altered pattern of reading circuit development was shown in the dyslexic group who, after the first 2 years of formal education, started to engage articulatory regions (the left inferior frontal gyrus and precentral gyrus) during an implicit reading task in fMRI^42^. Even though no group differences were observed early on, after two years children with dyslexia hypoactivated the left vOT and left IFG compared to both age and reading matched controls^42^. These observations are in line with the developmental model of the neural circuitry for reading^53^. This model suggests that in disrupted reading, instead of appropriate left vOT function development, compensatory shifts to inferior frontal sites supporting articulatory recoding together with engagement of right hemisphere regions is observed. Nonetheless, as the data from those studies cover only the first years of education, it is difficult to predict further development and whether activation in brain networks normalizes with further reading practice. For phonological skills acquired earlier than reading, the development of associated phonological networks was delayed in poor reading children. The bilateral STG, left middle temporal gyrus, right insula, and frontal cortex were engaged by typical readers during auditory rhyming at the beginning of formal education and by dyslexic readers two years later^48^. Similarly, a developmental delay in the functional connectivity of the reading network was suggested by the other study^43^. In contrast to typical readers showing decreasing connectivity from the left VOT to the IFG and PrCG, dyslexic readers at the age of 6 had significantly weaker connections, which then increased in strength at the age of 8 followed by downregulation and normalization from 8 years to 12 years^43^. Finally, the functional coupling between the left vOT and left IFG/STG prominent in typical reading children in first grade during processing audiovisual congruent nonword pairs showed a delayed development in poor reading children^44^.

ERP studies suggest a different development of visual N1 responses in implicit reading tasks between typical and dyslexic readers. In a study following children from second to fifth grade, individuals with dyslexia did not show the expected reduction in N1 word sensitivity observed in typical readers^38^. Group comparisons per time point showed that the N1 word sensitivity effect was reduced in children with dyslexia but this difference was no longer significant in fifth grade, where a trend for the opposite pattern was found. This seems to suggest delayed visual specialization maxima in dyslexic readers. In relation to phonological processing, another study^46^ reported attenuated LPC amplitudes in children with dyslexia compared to typical readers. Interestingly, those effects were confined to word stimuli (in contrast to pictures) and temporo-parietal electrode sites. While in typical readers LPC responses decreased after second grade, the opposite pattern was found in dyslexic readers. The results of these two studies are compatible with the notion of a protracted development of specialized responses in dyslexic readers. Along these lines, but less specific in terms of neural activity, an earlier report examined EEG power in three groups of 9-year-old children at two time points spaced by an interval of 2–3 years^56^. The results suggested more pronounced changes between sessions in spectral power (across multiple frequency bands) in poor compared to typical readers. This was also interpreted as indicative of a maturational delay in impaired readers. More recently, oscillatory activity was examined longitudinally in a population at risk for dyslexia at 5, 7, and 9 years of age in the context of auditory processing^57^. The study found an overall increase in sensitivity to phoneme-rate information (20 Hz) with reading onset, from 5 to 7 years of age. This change correlated negatively with reading and phonological skills and these auditory evoked responses were stronger in children that would develop dyslexia compared to typical readers after reading onset. These results suggest atypical neural synchronization in individuals with dyslexia to information at the temporal rate of phonemes in speech, which may be indicative of reading problems.

So far there is no conclusive evidence to determine whether the development of the reading pathways in dyslexia is altered or delayed. Studies on white matter connectivity provide support for protracted development of anatomical networks, while gray matter development has yet to be explored. Considering the evidence from functional studies examining the first two years of reading acquisition (shift from pre-reading to emergent reading) it seems that dyslexic children present an altered pattern of brain response when performing reading tasks. However, the few studies that go beyond the stage of emerging reading skills suggest that these functional alterations may mostly reflect a delay in the trajectory of reading network specialization. A delay rather than alteration was also observed in studies on phonological skills already in younger children. The dynamic interplay between phonological and reading skills appears to be a relevant factor for consideration in models of reading development in dyslexia.

## Neural precursors of reading

An important goal of neuroscientific studies of reading is to find early indicators of future performance to aid prevention and diagnosis. Only a few studies with an explicit focus on finding early markers of reading ability contain longitudinal brain data. Although they are not informative in terms of developmental brain dynamics, we provide a short summary in this section on some studies that used a single initial neuroimaging measurement to predict changes in reading and reading-related skills.

### Brain structure

A small-scale study focusing on gray matter properties found that pre-reading children who later developed dyslexia had a thinner cortex in several left-hemispheric regions: Heschl’s gyrus, lingual gyrus, medial frontal gyrus, middle cingulate gyrus, and the right orbito-frontal cortex. Most of those regions, identified as precursors of dyslexia outside the reading network, thickened over time in all children. The only exception was Heschl’s gyrus which was still significantly thinner in children with dyslexia at the age of 12^58^. Abnormalities of the left perisylvian region in dyslexia were also found in another recent study. Here, higher gyrification and variability of the folding of the left primary auditory cortex was found in children initially aged 6 and scanned again at the age of 8. At the pre-reading stage, streamline density (interpreted as the index of connectivity strength) of the left AF was significantly higher in dyslexic children than controls^59^. Regarding the left perisylvian region, increases in white matter volume in kindergarten were shown to be predictive of reading outcomes at the age of 8^60^. A similar predictive power of the left SLF developmental rate for reading was reported in another study^21^. Children who developed dyslexia, already before reading onset (aged 5–6), showed white matter alterations, i.e. lower FA of the left and right long segment of the AF^51^. In addition, FA of the left long segment of the AF contributed to the prediction of dyslexia on top of familial risk and cognitive predictors including phonological skills. Altogether these findings support the role of early left perisilvian cortex organization in dyslexia prediction, which could relate to post-mortem findings of neuronal migration anomalies around the sylvian fissure characterizing individuals with dyslexia^61^.

Several studies have aimed to predict reading development with neuroimaging techniques applied only at the pre-reading stage. For example, a recent study examined whether pre-reading neuroanatomical differences in cortical thickness and surface area can be related to dyslexia. This study showed that children who developed dyslexia were characterized by a reduced surface area in the bilateral fusiform gyri, while no differences were observed for cortical thickness^62^. Since the surface area is more related to prenatal influences, in contrast to cortical thickness showing plastic changes throughout development^12^, this finding speaks for predominantly prenatal anomalies in dyslexia. A few other studies investigated predictors of reading development related to white matter properties. It was shown that pre-reading children with a family risk of dyslexia compared to their peers had higher T1 intensities of the left AF which were interpreted as reduced myelin concentration. This measure was also a significant predictor of dyslexia (explaining 47% of variance), while the best predictive model including both behavioral measures (IQ, phonological representations, working memory) and T1 intensities reached 80% accuracy. The effect, however, was not found for the most commonly assessed FA measure^63^. Nonetheless, other studies reported that FA of reading-related tracts can serve as an important predictor of reading development. For example, FA of the left AF, left and right superior longitudinal fasciculus (SLF), and left inferior cerebellar peduncle in 6-year olds was shown to make a unique contribution to reading outcome two years later^64^. Additionally, high FA of the posterior right SLF in pre-readers at risk of dyslexia, among other factors like high socioeconomic background and speech production accuracy, was identified as protective, leading to the development of typical reading skills^65^. Also in the wider age range (children aged 8–14, with a time gap of 1.5–4 years) higher FA of the direct frontotemporal segment of the left AF predicted progress in reading^66^. This set of findings suggests that white matter alterations in the reading networks predate reading onset and are good candidates for the predictors of dyslexia and reading development in general.

Certainly, the above-mentioned studies require replication of results across independent longitudinal studies to increase their confidence due to low sample sizes and, as a result, low statistical power. Even though longitudinal studies on brain structure in dyslexia are still scarce, it is suggested that structural abnormalities (especially of the left perisylvian cortex and left AF) present before reading onset are good candidates for markers to be used as predictors of dyslexia. Nonetheless, we conclude that both early gray and white matter abnormalities may indicate challenging reading development.

### Brain function

Functional studies have related phonological and visual processing with future reading outcomes. Specifically, learning to read consists in creating abstract representation of written words and connecting it to areas coding for meaning and pronunciation^67^. In line, in fMRI studies, the extent of print-speech convergence in the left STG, a region also highlighted by structural studies as crucial for reading acquisition, could predict reading performance achieved one^68^ or two years later^69^ in beginning English readers. Co-activation in the left STG during speech and print processing also differentiated young readers from pre-readers and correlated with reading performance in Polish six-year olds^70^. Improvement of reading skills over time was also predicted by changes in the functional connectivity patterns within the reading network, with stronger connections between STG and IFG during a written word rhyming task^71^. Moreover, in the phonological task the functional connection strength at the pre-reading stage between the left IPC, left IFG, left vOT, and right angular gyrus predicted later reading development^49^.

Children having familial risk diagnosed with dyslexia at the end of the second grade already had underactived left vOT for letters and false fonts in kindergarten (age 5.5) when compared to typically developing children without the familial risk of dyslexia^72^. It was also shown that reading gains in teenagers with dyslexia could be accurately predicted by combining information about functional activation in the right IFG during a phonological task with information about the strength of white matter integrity in the right SLF and AF. Since this pattern was present only in children with dyslexia, but not in controls, it was interpreted as a brain mechanism altered and specific for dyslexia rather than reflecting growth in reading ability in general^73^. Right IFG hyperactivity during a phonological task at the pre-reading stage was present only in those children at risk of dyslexia who developed typical reading skills, but not in children developing dyslexia, which suggests a protective mechanism^74^.

Several EEG studies used electrophysiological responses in pre-reading stages to predict later language skills. ERP indices of phonological processing recorded as early as a few days after birth discriminated between children with and without familial risk for dyslexia and correlated with phonological and naming skills and letter knowledge at 6.5 years^75^. More specific to reading, a study used phonology-related ERP responses from newborns to classify children into dyslexic, poor, and typical readers based on their reading skills eight years later and achieved 81.25 % correct classification^76^. In another study, auditory ERPs in an oddball paradigm in six-month old infants predicted 44% of variability in reading speed at 14 years and improved prediction from cognitive assessments in preschool^77^. Similar findings were reported for brain potentials related to late detection of phoneme changes in kindergartners^78^. The ERPs significantly improved the prediction of future reading skills combined with behavioral measures in second and third grade and were the only significant predictor of reading skills in fifth grade. Altogether, this evidence suggests that at least some profiles of impaired readers may present very early differences in auditory processing. Several studies showed that ERPs of visual print processing, in combination with fMRI activations, in kindergarten could have an added value in identifying future poor readers^37,79,80^. In addition, aside from these specific auditory and visual responses, general oscillatory activity in resting-state EEG measured by spectral power at the age of 3 years, also discriminated between children at risk that would be classified as good and poor readers in the third grade^81^. In that study, spectral amplitudes in lower delta and lower alpha frequency bands correlated positively and negatively, respectively, with reading and rapid naming performance.

In general, these studies suggest that prediction of dyslexia can be improved when neuroimaging measures are taken into consideration in addition to behavioral precursors of reading. At the same time, there is little overlap between the potential neuroanatomical predictors, perhaps influenced by the small number of studies and usually low sample sizes. Functional predictors seem more promising. Specifically, infant ERP responses to auditory processing, print sensitivity of the left vOT in pre-reading children, and the extent of print-speech convergence in the left STG show some consistency. However, low sample sizes and inadequate statistical models (e.g. feature selection bias and overfitting in machine learning) are still major obstacles in the field of single-subject clinical prediction with neuroimaging techniques^82^.

## Longitudinal design in reading research—challenges and future directions

Most valuable longitudinal neuroimaging studies of dyslexia are conducted for relatively long time periods, tracking children from the pre-reader to skillful reader stage. At the same time, such studies are particularly difficult to carry out. Since only 10% of children will eventually be diagnosed with dyslexia, the initial population must be enriched with children at familial risk of becoming dyslexic, thereby severely constraining recruitment. This, however, only increases the chances of having selected a child that will eventually be diagnosed with dyslexia to about 40%. This still does not ensure large enough statistical power in the final sample due to the challenges inherent in performing neuroimaging research on children (e.g., recruitment, compliance, head movement), especially those young enough not to have learned to read. As children must be followed for several years (at least 2 or 3) in order to receive a diagnosis of dyslexia, the risk of attrition increases substantially. To minimize drop out at each stage, families should receive feedback on children’s performance whenever possible and should be informed about the outcomes of the study. Ideally, longitudinal data should also include additional information about the amount and type of reading instruction (which varies depending on the educational system and may differ across research centers), print exposure (measured with home literacy environment) and, finally, whether special training or reading interventions took place in the period of the study. These factors are likely to have an important impact on the course of neural specialization for reading and constitute a source of individual variability which is not typically addressed in the literature. This issue may become particularly relevant in studies pooling data from multiple centers or linguistic populations. Combining data from different laboratories in a multi-site approach is a good solution for increasing sample sizes and, as a result, statistical power and reliability of findings. Hence an important challenge would be generalization across languages. For example, a recent study investigated the reproducibility and generalizability of EEG measures related to speech sound discrimination using multi-site data from four different orthographic languages^83^. The study provided reassuring evidence for the capacity of a passive oddball paradigm to capture brain responses related to speech and non-speech sound discrimination with high reproducibility across languages. However, the findings demonstrated important inconsistencies in the reported differences between reading groups. Thus, longitudinal studies may also try to address these differences and consider language-specific elements, or strategies for reading instruction, when hypothesizing developmental trajectories of specific brain systems.

In conclusion, this review summarizes the current state of knowledge of the developmental trajectory in typical and atypical reading development as revealed by longitudinal studies. Thanks to the longitudinal approach, we have learned that age or reading experience effects do not always show positive linear trends as sometimes implied in cross-sectional studies^84–86^. Longitudinal studies have also demonstrated that at the beginning of reading acquisition, dyslexic readers present an altered pattern of brain sensitivity to print, possibly followed by delayed specialization in later years. Early prediction of dyslexia might be improved with neuroimaging measures, and several candidate markers have been appointed. Future longitudinal studies, preferably with larger sample sizes, will help us clarify their reliability.

## Supplementary information

Supplementary Data Set 1

## Data Availability

Data sharing not applicable to this article as no datasets were generated or analyzed.

## Code availability

No code was generated during the current study.
